# Retraction Note: Relationship between emotional intelligence and job well-being in Chinese clinical nurses: multiple mediating effects of empathy and communication satisfaction

**DOI:** 10.1186/s12912-025-02920-5

**Published:** 2025-03-10

**Authors:** Xue Li, Hongjuan Chang, Quanying Zhang, Jianli Yang, Rui Liu, Yajie Song

**Affiliations:** 1 College of Nursing, Xinxiang Medical University, No.601 Jinsui Avenue, Hongqi District, Xinxiang City, Henan Province 453003 China; 2 Nursing Department, The First Affiliated Hospital of Xinxiang Medical University, Henan Province, Xinxiang City, 453100 China

**Retraction Note: BMC Nursing (2021) 20:144**

10.1186/s12912-021-00658-4

The Editors have retracted this article. Following the publication of this article, concerns were raised regarding the use of the copyrighted Jefferson Scale of Empathy (JSE) without proper permission. Despite the Publisher’s several attempts to contact the authors regarding the issue, they were unable to get any response from the authors.

None of the authors have responded to any correspondence from the editor or publisher about this retraction.

